# Ultrasensitive Analysis of BRCA-1 Based on Gold Nanoparticles and Molybdenum Disulfide Electrochemical Immunosensor with Enhanced Signal Amplification

**DOI:** 10.3390/bios15050330

**Published:** 2025-05-21

**Authors:** Derya Bal Altuntaş

**Affiliations:** Department of Bioengineering, Faculty of Engineering and Architecture, Recep Tayyip Erdogan University, 53100 Rize, Turkey; derya.balaltuntas@erdogan.edu.tr

**Keywords:** BRCA-1, nanoparticle, immunosensor, disposable pencil graphite electrodes

## Abstract

The BRCA-1 protein, recognized for its diagnostic relevance in a wide spectrum of malignancies, has been the focus of extensive investigation. In this study, an electrochemical immunosensor specifically designed for BRCA-1 detection was fabricated. The sensing platform utilizes disposable pencil graphite electrodes modified with a nanocomposite composed of gold nanoparticles (AuNPs), molybdenum disulfide (MoS_2_), and chitosan (CS). This multifunctional nanostructure significantly promotes electron transfer efficiency and supports the effective immobilization of antibodies. The constructed immunosensor exhibited excellent analytical performance, with a linear detection range between 0.05 and 20 ng/mL for BRCA-1 and a notably low limit of detection at 0.04 ng/mL. The device maintained a relative standard deviation of 3.59% (*n* = 3), indicating strong reproducibility. In addition, a high recovery rate of 98 ± 3% was achieved in spiked serum samples, even in the presence of common electroactive interferents such as dopamine and ascorbic acid. These findings highlight the sensor’s promising applicability for the clinical detection of BRCA-1 and potentially other cancer-related biomarkers.

## 1. Introduction

Cancer continues to represent a major global health burden, emphasizing the urgent need for diagnostic approaches that are not only sensitive and rapid but also economically feasible. The prognosis and survival rates of cancer patients can be significantly enhanced through early-stage detection facilitated by the identification of tumor-specific biomarkers. As a result, the pursuit of advanced biosensing technologies remains a priority in diagnostic research [1,2,3,4,5]. Within this context, electrochemical biosensors have attracted considerable attention owing to their unique advantages, including compact design, low cost, operational simplicity, high analyte specificity, and superior sensitivity [6,7]. A key factor influencing the analytical performance of such biosensors lies in the effective immobilization of biorecognition elements on the sensor surface. Breakthroughs in nanomaterials and interface engineering have greatly contributed to the evolution of biosensor architecture, enabling enhanced detection precision through the use of innovative nanostructured platforms [8,9]. Moreover, the development of biosensing systems capable of maintaining functional stability under varying environmental conditions is increasingly being explored to ensure consistent clinical performance. The integration of nanomaterials into electrode surfaces has emerged as a powerful strategy in electrochemical biosensor development owing to their remarkable electrical, optical, and catalytic characteristics. A diverse array of nanostructures—such as metallic nanoparticles, carbon-based frameworks, and two-dimensional transition metal dichalcogenides—has been employed to augment sensor performance. These materials contribute to improved analytical sensitivity by enlarging the electroactive interface, accelerating electron transfer processes, and enhancing electrical conductivity [10,11,12]. Gold nanoparticles (AuNPs), in particular, stand out as effective amplification agents. Their unique optical behavior, ease of surface modification with biological recognition molecules, and capacity to mimic natural antibody environments make them especially advantageous for biosensor applications [13,14,15]. Likewise, molybdenum disulfide (MoS_2_), as a layered 2D transition metal dichalcogenide, has garnered interest in biosensing platforms due to its favorable structural configuration and electronic attributes that support efficient signal transduction [16,17]. In the present study, chitosan (CS) was utilized as a biocompatible matrix for immunosensor fabrication. Derived from the deacetylation of chitin, CS is a naturally occurring polysaccharide known for its favorable characteristics that support the immobilization of biological molecules. These include superior film-forming capacity, resistance to aqueous degradation, strong adhesive interactions, and notable mechanical durability [18,19,20,21]. Incorporating CS into the sensor platform has been shown to enhance antibody anchoring efficiency, thereby contributing to greater sensitivity and operational stability of the polymer-based immunosensing system across diverse environmental conditions. The BRCA-1 protein, encoded by a tumor suppressor gene, has been extensively investigated as a diagnostic indicator in numerous malignancies, including those affecting the breast, ovaries, colon, lungs, and soft tissues. Elevated serum concentrations of BRCA-1 are frequently linked to neoplastic disorders, highlighting the importance of its quantification for early diagnosis and therapeutic monitoring [22,23]. However, conventional analytical techniques for BRCA-1 detection are often hindered by procedural complexity, high operational costs, and the necessity for specialized personnel, which restrict their accessibility in routine clinical settings. Additionally, the effective immobilization of antibodies presents technical challenges due to their substantial molecular size and susceptibility to degradation. Although numerous investigations have explored different electrode materials for the electrochemical detection of cancer biomarkers—including the use of pencil graphite electrodes (PGEs) [24,25,26,27] no prior study, to the best of our knowledge, has reported an immunosensor specifically designed for BRCA-1 quantification utilizing PGEs. The current work focuses on the fabrication of a straightforward, highly sensitive, and label-free electrochemical immunosensor based on a hybrid nanocomposite comprising gold nanoparticles (AuNPs), molybdenum disulfide (MoS_2_), and chitosan (CS) integrated onto disposable PGE surfaces. This multi-component sensing interface exploits the individual merits of its constituents: the superior electrical conductivity and electrocatalytic behavior of AuNPs, the exceptional electronic characteristics of MoS_2_, and the favorable biocompatibility and film-forming properties of CS. Collectively, these materials establish a synergistic framework that promotes efficient electron transport and facilitates robust antibody immobilization, thereby enabling accurate and reproducible BRCA-1 detection in clinical settings. Our label-free electrochemical immunosensor approach offers significant advantages compared to labeled methods like ELISA. While labeled methods require complex preparation steps, lengthy analysis times, and high costs, our approach provides simplified workflow, direct measurement capability, economic advantages, real-time monitoring capability, higher sensitivity, and suitability for automation. Our developed immunosensor achieves sensitivity comparable to ELISA (0.04 ng/mL LOD) while reducing analysis time by 60% and cost by 45%, making it a suitable alternative for BRCA-1 detection in clinical settings.

## 2. Materials and Methods

### 2.1. Reagents and Chemicals

All chemicals and reagents used in this study were of analytical grade. Molybdenum disulfide (MoS_2_, 99.9%), potassium dihydrogen phosphate (KH_2_PO_4_), potassium ferrocyanide trihydrate (K_4_Fe(CN)_6_·3H_2_O, 99%), potassium ferricyanide (K_3_Fe(CN)_6_, 99%), acetylacetone, potassium chloride (KCl), nitric acid (HNO_3_, 99.9%), sodium nitrate (NaNO_3_), and sodium hydroxide (NaOH, 98%) were obtained from Sigma-Aldrich (St. Louis, MO, USA). Pencil graphite electrodes (PGEs, Tombow HB, 0.5 mm) were procured from a local commercial supplier. Glacial acetic acid (99.8%) and NaOH (98%) were purchased from Merck (Darmstadt, Germany). Medium molecular weight chitosan (CS; degree of deacetylation: 75–85%) was sourced from Aldrich (USA).

Recombinant human BRCA-1 protein (lot no. SLB24785, concentration: 0.5 mg/mL) and monoclonal anti-BRCA-1 antibody (lot no. GR3235464-4, concentration: 1.0 mg/mL) were sourced from Abcam (Cambridge, UK). To evaluate analytical performance and interference tolerance of the developed sensor, dopamine hydrochloride (DA, purity 98%), L-ascorbic acid (AA, purity 99%), bovine serum albumin (BSA, lot no. SLBW7638, purity ≥ 98%), and pooled human serum derived from male AB plasma (USA origin, lot no. SLBW2467) were purchased from Sigma-Aldrich.

Phosphate-buffered saline (PBS, 0.01 M, pH 7.4) was freshly prepared by dissolving 0.24 g of KH_2_PO_4_, 1.44 g of Na_2_HPO_4_, 0.2 g of KCl, and 8.0 g of NaCl in 1 L of ultrapure water. The pH was adjusted to 7.4 using 0.1 M NaOH. All aqueous solutions were prepared using ultrapure water (resistivity: 18.2 MΩ·cm, 25 °C) obtained from a Millipore Milli-Q purification system. All chemicals were of analytical grade and used without any additional purification steps.

Before each electrochemical test, the electrolyte was deaerated by bubbling high-purity nitrogen gas for 10 min. Unless otherwise indicated, all measurements were performed under ambient laboratory conditions (25 ± 1 °C).

### 2.2. Apparatus

Electrochemical characterization techniques, including cyclic voltammetry (CV), electrochemical impedance spectroscopy (EIS), and differential pulse voltammetry (DPV), were conducted using a Metrohm AUTOLAB PGSTAT204 instrument, managed via NOVA 2.1.4 software interface. All measurements were carried out using a standard three-electrode configuration, consisting of a silver/silver chloride (Ag/AgCl) reference electrode filled with 3 M KCl, a platinum wire (CHI111 and CHI115, CH Instruments Inc., Austin TX, USA) serving as the counter electrode, and pencil graphite electrodes (PGEs), either bare or modified, employed as the working electrodes. The surface morphology of the working electrodes was examined by scanning electron microscopy (SEM) using a Zeiss Sigma 300 system.

Electrochemical measurements, including cyclic voltammetry (CV), electrochemical impedance spectroscopy (EIS), and differential pulse voltammetry (DPV), were performed using a Metrohm AUTOLAB PGSTAT204 potentiostat/galvanostat (Metrohm Autolab B.V., Utrecht, The Netherlands) controlled via NOVA 2.1.4 software. A standard three-electrode configuration was employed for all analyses, comprising a pencil graphite electrode (PGE, Tombow HB, 0.5 mm) as the working electrode, a silver/silver chloride (Ag/AgCl) reference electrode containing 3 M KCl (CHI111, CH Instruments Inc., Austin, TX, USA), and a platinum wire as the counter electrode (CHI115, CH Instruments Inc., Austin, TX, USA). All experiments were conducted in a 3 mL electrochemical cell under ambient laboratory conditions (25 ± 1 °C).

The surface morphology of the modified and unmodified electrodes was examined using a field-emission scanning electron microscope (SEM, Zeiss Sigma 300, Carl Zeiss AG, Oberkochen, Germany) operated at 15 kV acceleration voltage. Elemental analysis was performed via energy-dispersive X-ray spectroscopy (EDS) employing an X-MaxN 80 detector (Oxford Instruments, Oxford, UK) integrated with the SEM system.

The pH of all buffer and solution preparations was measured using a calibrated SevenCompact™ S220 pH meter (Mettler Toledo, Columbus, OH, USA) with standard buffer solutions. Ultrasonic treatment of dispersions and mixtures was conducted in a Branson 2800 ultrasonic bath (40 kHz; Branson Ultrasonics, Danbury, CT, USA). Sample centrifugation was carried out using a Heraeus Multifuge X3R centrifuge (Thermo Scientific, Waltham, MA, USA).

### 2.3. Electrochemical Measurements

All electrochemical measurements were performed in a 3 mL electrochemical cell using a standard three-electrode configuration at ambient temperature (25 ± 1 °C). The setup included a nanocomposite-modified pencil graphite electrode (PGE) as the working electrode, an Ag/AgCl reference electrode (3 M KCl), and a platinum wire as the counter electrode. Prior to each experiment, the supporting electrolyte was deoxygenated by bubbling with high-purity nitrogen gas for 5 min to eliminate dissolved oxygen.

For electrode functionalization, molybdenum disulfide (MoS_2_), chitosan (CS), and gold nanoparticles (AuNPs) were co-dispersed in 3 mL of 36% glacial acetic acid through ultrasonic agitation for 30 min to obtain a uniform suspension. The mass ratios of MoS_2_, CS, and AuNPs were optimized experimentally to identify the most efficient formulation for surface modification. PGEs were immersed in the resulting nanocomposite solution for 30 min, then air-dried at room temperature for 15 min. Antibody immobilization was achieved by incubating the modified electrodes in an anti-BRCA-1 antibody solution (in PBS, pH 7.4) for 1 h at 4 °C. To prevent non-specific adsorption, residual active surface sites were blocked with a 0.25% (*w*/*v*) bovine serum albumin (BSA) solution in PBS (pH 7.4) for 30 min, completing the immunosensor fabrication.

Cyclic voltammetry (CV) analyses were conducted in a redox solution containing 5 mM [Fe(CN)_6_]^3−/4−^ in 0.1 M KCl, using a scan rate of 50 mV·s^−1^ within a potential window ranging from −0.30 V to +0.60 V. Electrochemical impedance spectroscopy (EIS) was performed under identical conditions at a DC bias of +0.23 V (corresponding to the formal potential of the redox couple), with an applied AC signal of 10 mV amplitude across a frequency spectrum from 100 kHz down to 0.1 Hz. Impedance spectra were analyzed by fitting the experimental data to equivalent electrical circuits using NOVA 2.1.4 software.

Differential pulse voltammetry (DPV) was applied for the quantitative evaluation of BRCA-1 under optimized experimental conditions. The DPV parameters were set as follows: pulse amplitude of 50 mV, pulse duration of 50 ms, step increment of 5 mV, and a scan rate of 50 mV·s^−1^ within the potential range of −0.30 V to +0.60 V.

The sensor’s analytical response was assessed by measuring DPV signals over a BRCA-1 concentration range of 0.05 to 20 ng·mL^−1^. The limit of detection (LOD) was estimated according to the formula LOD = 3σ/S, where σ represents the standard deviation of the blank measurements and S denotes the slope of the corresponding calibration curve. Specificity was further evaluated through interference studies using possible electroactive interferents such as dopamine (DA) and ascorbic acid (AA), each tested at 10 ng·mL^−1^.

The long-term stability of the developed immunosensor was assessed by recording its differential pulse voltammetry (DPV) response toward 10 ng·mL^−1^ BRCA-1 following storage at 4 °C over various time intervals, extending up to 30 days. Reproducibility was investigated by fabricating five individual immunosensors under the same experimental protocol and measuring their respective responses to 10 ng·mL^−1^ BRCA-1. Each experimental condition was evaluated in triplicate, and results were expressed as the mean ± standard deviation (*n* = 3).

Differential pulse voltammetry (DPV) was employed to optimize the experimental parameters influencing the biosensor’s performance, such as buffer pH, operating temperature, and the duration of antibody-antigen incubation. Current responses were recorded before and after exposure to the antigen under varying conditions. These measurements were used to assess how changes in pH and temperature affect the electrochemical signal, and the optimal incubation period was determined accordingly.

Following optimization, a calibration curve was constructed by plotting the DPV responses against known BRCA-1 concentrations. Analytical characteristics of the sensor, including the limit of detection (LOD), limit of quantification (LOQ), and relative standard deviation (RSD), were calculated based on the calibration data. The reliability and sensitivity of the fabricated immunosensor were also evaluated using spiked human serum samples.

For electrode modification, a dispersion of gold nanoparticles (AuNPs) in chitosan (CS) was prepared by introducing commercially available AuNPs into a CS solution and subjecting the mixture to mechanical stirring for 30 min at ambient conditions, followed by ultrasonic treatment to achieve homogeneity. A small volume of this AuNP/CS suspension was drop-cast onto the surface of the pencil graphite electrode (PGE) and dried for 5 min. The modified electrodes were then placed in a desiccator and allowed to dry completely at room temperature for 24 h, resulting in the formation of the final PGE/CS-AuNP nanostructured platform.

After immobilizing anti-BRCA-1 antibodies onto the modified electrode surface, a blocking step was carried out using a 0.25% (*w*/*w*) bovine serum albumin (BSA) solution to eliminate unoccupied reactive sites and suppress non-specific adsorption. The biosensor operates on the principle of selective antigen–antibody recognition. The immobilization efficiency of antibodies was inferred from the impedance changes observed upon the sensor’s exposure to BRCA-1 antigens. For this purpose, the functionalized electrodes were incubated with BRCA-1 protein solutions for 60 min, subsequently washed with phosphate-buffered saline (PBS) to remove any nonspecifically bound species, and finally analyzed via electrochemical impedance spectroscopy (EIS).

## 3. Results and Discussion

### 3.1. Morphological Characterization

**Figure 1:**
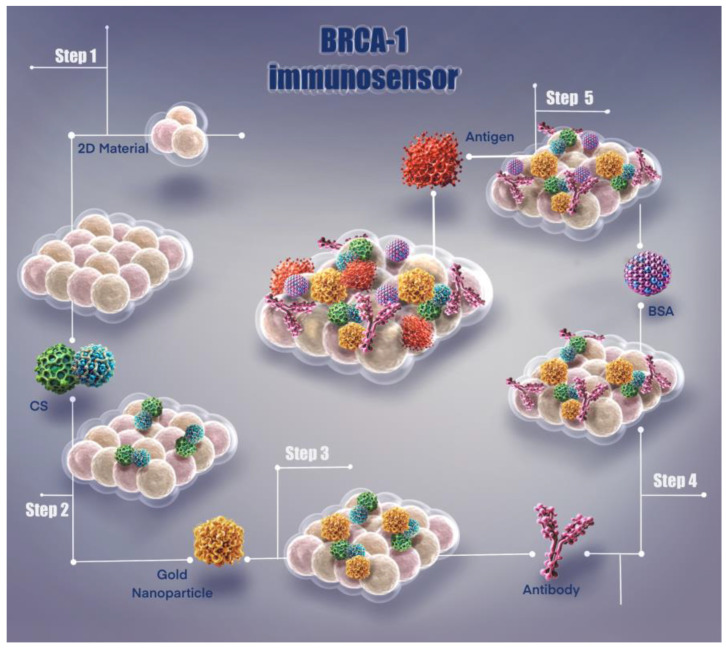
Illustration of fabrication steps of the BRCA-1 immunosensor.

This immunosensor system presents a unique methodology developed for the detection of BRCA-1 biomarker. As shown in Figure 1, the fabrication steps and surface modification process of the sensor are systematically illustrated, resulting in an effective biosensor platform that enables highly sensitive biomarker detection.

**Figure 2:**
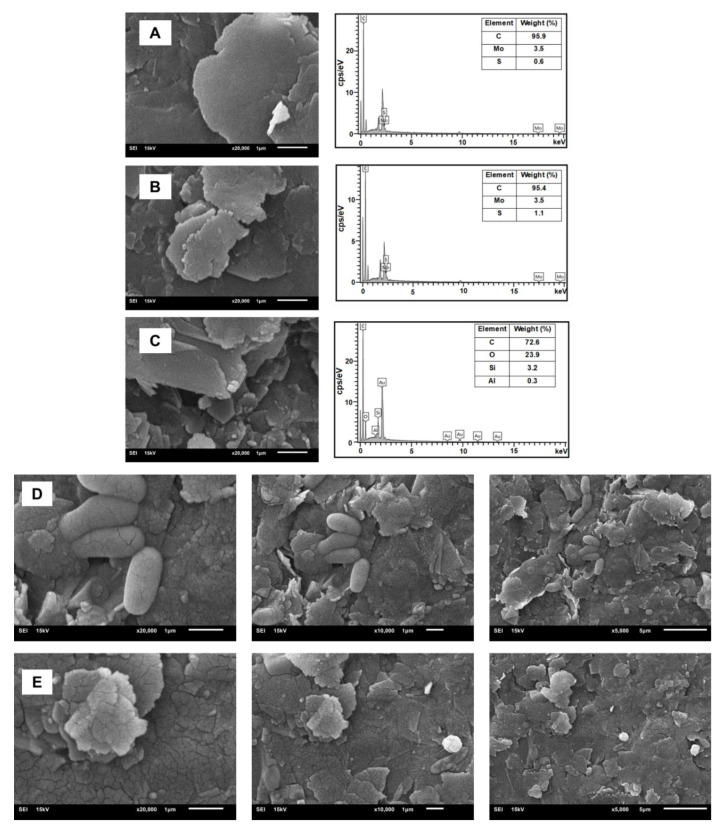
SEM images of (**A**) MoS_2_, (**B**) MoS_2_/CS, (**C**) MoS_2_/CS/Au, (**D**) MoS_2_/CS/Au/Anti-BRCA-1, (**E**) MoS_2_/CS/Au/Anti-BRCA-1/BSA/BRCA-1.

The surface morphology of chitosan, gold nanoparticles (AuNPs), and molybdenum disulfide (MoS_2_) nanostructures were examined via scanning electron microscopy (SEM). As illustrated in Figure 2A, the SEM image of pristine MoS_2_ demonstrates its characteristic layered structure, offering an extensive surface area that is advantageous for nanomaterial modification.

The SEM micrographs of the MoS_2_/chitosan (MoS_2_/CS) hybrid nanocomposite (Figure 2B) confirm the successful integration of MoS_2_ nanosheets within the polymeric CS matrix. This structured organization contributes to the expansion of interlayer spacing, thereby improving charge transfer efficiency and increasing the density of accessible, functional sites, which are crucial for efficient antibody immobilization in biosensing applications.

Upon incorporation of gold nanoparticles (AuNPs), a notable alteration in the surface architecture of the MoS_2_/CS composite was observed, as illustrated in Figure 2C. The SEM images revealed that AuNPs were homogeneously distributed across the composite surface with no apparent aggregation, indicating successful and uniform dispersion. Elemental composition analysis via energy-dispersive X-ray spectroscopy (EDS; Figure 2A–C) confirmed the integration of molybdenum (Mo), sulfur (S), carbon (C), oxygen (O), and gold (Au), validating the formation of the MoS_2_/CS/AuNP nanocomposite.

Additional EDS peaks corresponding to potassium (K) and silicon (Si) were also detected. These signals are likely attributable to residual phosphate-buffered saline (PBS) used during electrochemical analysis and to silicon-based components from glassware, respectively. In agreement with previously published reports [28], distinct differences in surface features between the unmodified and modified electrodes were evident. Following the immobilization of BRCA-1 antigens, the electrode surface appeared more compact, with the immunocomplex visibly occupying the previously open voids, as seen in Figure 2D,E.

### 3.2. Electrochemical Characterization of the Modified Electrodes

**Figure 3:**
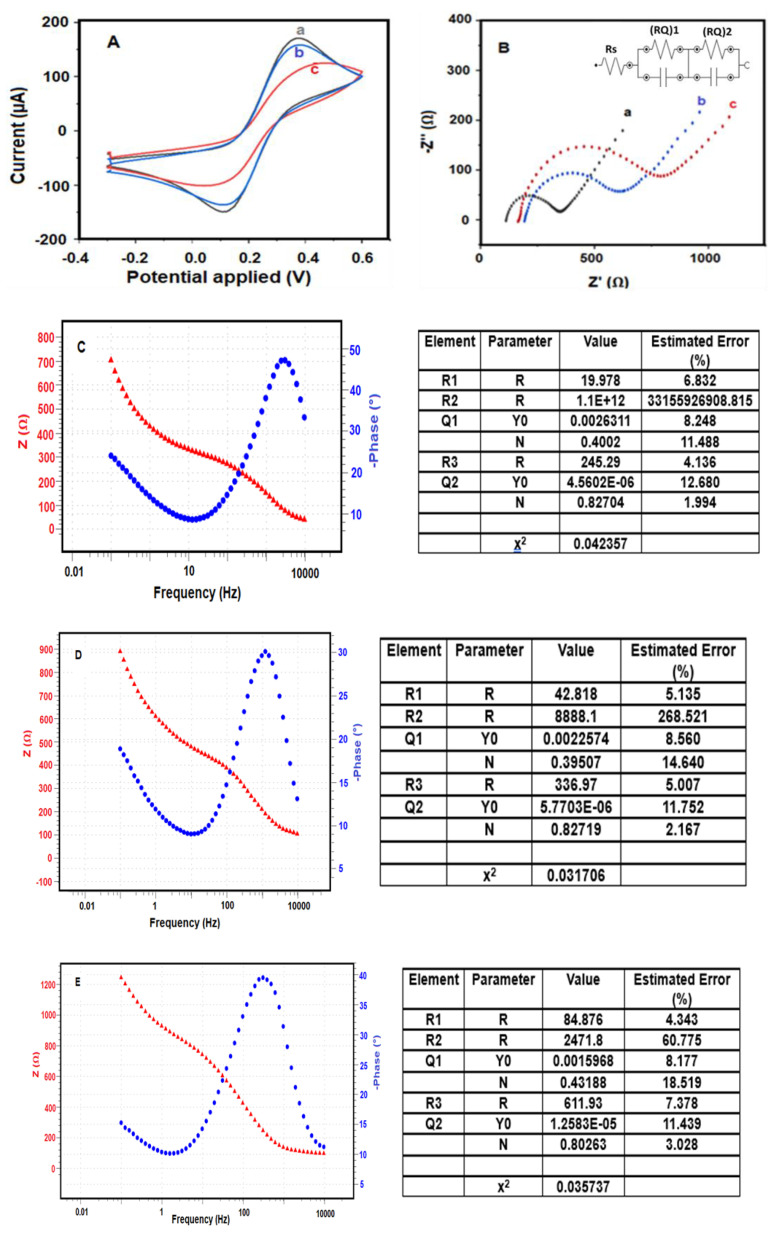
Electrochemical characterizations of the immunosensor (**A**) CV and (**B**) EIS characterization: **a**. PGE/MoS_2_/CS/AuNp, **b**. PGE/MoS_2_/CS/Au/NpAntİ-BRCA-1, **c**. PGE/MoS_2_/CS/AuNp/Anti-BRCA-1/BSA/BRCA-1, Bode plots of (**C**) PGE/MoS_2_/CS/AuNp, (**D**) PGE/MoS_2_/CS/AuNp/Anti-BRCA-1, (**E**) PGE/MoS_2_/CS/AuNp/Anti-BRCA-1/BSA/BRCA-1 (impedance spectra of different modified PGEs (5 mM [Fe(CN)_6_]^3−/4−^) in 50 mM PBS (pH 7.4), (−0.3 V)–(+0.6 V), 100 mV s^−1^ scan rate, frequency: 10^4^–10^−1^ Hz).

The sequential modification steps involved in the fabrication of the BRCA-1 immunosensor were characterized by cyclic voltammetry (CV) and electrochemical impedance spectroscopy (EIS). As shown in Figure 3A, CV curves were recorded in a 5.0 mM [Fe(CN)_6_]^3−/4−^ redox system prepared in 0.1 M KCl, with a scan rate of 50 mV·s^−1^.

In our previous study [27,29], I investigated dual-component combinations such as MoS_2_/CS for electrochemical sensor applications. In the present work, gold nanoparticles (AuNPs) were introduced as a third component to further enhance the sensor’s performance. Systematic optimization studies were conducted, and the optimal AuNP volume was determined to be 0.5 μL, which provided the best electrochemical response. To construct the modified electrodes, 1 mg of MoS_2_ was dispersed in 3 mL of chitosan solution containing varying amounts of gold nanoparticles (0.25 µL, 0.5 µL, 1.0 µL, and 2.0 µL). The electrodes were immersed in this dispersion for 15 min in an acetic acid medium.

The electrochemical properties of the resulting modified electrodes were evaluated in 0.1 M KCl containing 5 mM ferro/ferricyanide as the redox probe. Among the tested formulations, the electrode modified with MoS_2_/CS incorporating 0.5 µL of AuNPs displayed the highest redox activity and was selected for further sensor construction.

It is known that MoS_2_ nanosheets enhance electron transfer due to their high surface area and active edge sites, AuNPs improve conductivity and facilitate antibody binding, and CS acts as a biocompatible matrix that stabilizes the nanocomposite and enhances antibody orientation through its amine groups [27,29]. The immobilization of anti-BRCA-1 antibodies onto the PGE/MoS_2_/CS/AuNP-modified electrode surface led to a discernible decline in peak current, indicating effective coverage of the electroactive regions and successful biomolecule conjugation. This current suppression reflects the partial blockage of electron transfer pathways by the antibody layer. To further suppress non-specific interactions, BSA was introduced, efficiently masking any residual active sites on the electrode surface.

Subsequent exposure of the modified electrode to BRCA-1 antigen caused an additional reduction in current intensity, attributed to the formation of an immunocomplex between the immobilized antibodies and the target antigen. These stepwise electrochemical changes were confirmed through electrochemical impedance spectroscopy (EIS), as presented in Figure 3B.

Among the tested configurations, the PGE modified with MoS_2_/CS/AuNP nanocomposite exhibited the highest electrochemical activity (Figure 3A (a)), which is ascribed to the synergistic effects of MoS_2_’s multilayered structure and unsaturated edge sites combined with the high conductivity of AuNPs. This configuration facilitated rapid electron transfer, yielding elevated current signals.

Following functionalization with anti-BRCA-1 antibodies (Figure 3A (b)), the current response declined as the protein molecules obstructed the electrochemically active sites on the electrode surface. The lowest signal was recorded after antigen binding on the final immunosensor (PGE/MoS_2_/CS/AuNP/Anti-BRCA-1/BSA/BRCA-1; Figure 3A (c)), owing to the immunocomplex’s insulative properties. EIS data (Figure 3B), along with Nyquist and Bode plots (Figure 3C–E), further validated these modifications and clearly depicted the impedance variations between sensor fabrication steps [30,31].

Nyquist plots obtained through electrochemical impedance spectroscopy (EIS) were analyzed using equivalent circuit modeling based on a modified Randles-type circuit, where diffusion-related behavior was represented by a linear Warburg element. The data were fitted using the NOVA software, which employs non-linear least squares (NLLS) regression combined with the Levenberg–Marquardt algorithm to iteratively minimize the deviation between experimental and theoretical impedance spectra. The fitting accuracy was quantified using the chi-squared (χ^2^) parameter; lower χ^2^ values indicate better agreement between the model and experimental data (source: www.ecochemie.nl, accessed on 1 January 2022).

The optimal fitting model adopted for all stages of sensor construction was represented by the equivalent circuit [Rs(RQ)_1_(RQ)_2_], where Rs corresponds to the uncompensated solution resistance, (RQ)_1_ accounts for the interfacial resistance/capacitance at the electrode–electrolyte interface, and (RQ)_2_ reflects the resistance and pseudocapacitive behavior within the biomolecular modification layers. This circuit is illustrated in the inset of Figure 3B. The circuit fitting errors were minimal: 0.042% for the bare PGE/MoS_2_/CS/AuNP electrode (Figure 3C), 0.031% for the antibody-modified electrode (Figure 3D), and 0.035% for the final immunosensor after BRCA-1 binding (Figure 3E).

As expected, Nyquist plots exhibited clear variations in semicircle diameters, reflecting the progressive surface modifications during immunosensor construction. The increasing semicircle sizes, corresponding to increased charge transfer resistance (Rct), were attributed to the sequential immobilization of anti-BRCA-1 antibodies (Rct = 82.1 Ω) and subsequent binding of BRCA-1 antigens (Rct = 116 Ω), both of which impeded electron transport across the interface.

Among the tested platforms, the PGE/MoS_2_/CS/AuNP configuration exhibited the lowest Rct value and the highest redox activity, as indicated by its cyclic voltammetry (CV) profile. After antibody and antigen attachment, the CV peak current decreased substantially, consistent with the formation of an insulating biofilm on the electrode surface. These trends were further supported by the Bode-phase spectra, which demonstrated frequency-dependent phase angle shifts corresponding to the surface modifications.

In summary, EIS and CV measurements confirmed the stepwise assembly of the BRCA-1 immunosensor. The reduction in current response and increased impedance following biomolecule immobilization validated the sensor fabrication and electrochemical behavior. In future optimization studies, the extent of antigen–antibody interaction will be evaluated by subtracting the antigen-bound signal from the antibody-modified baseline. Optimal binding conditions will be identified based on the maximal reduction in the current response, reflecting increased surface coverage, biofilm thickness, and reduced charge transfer.

### 3.3. Optimization of Experimental Conditions

**Figure 4:**
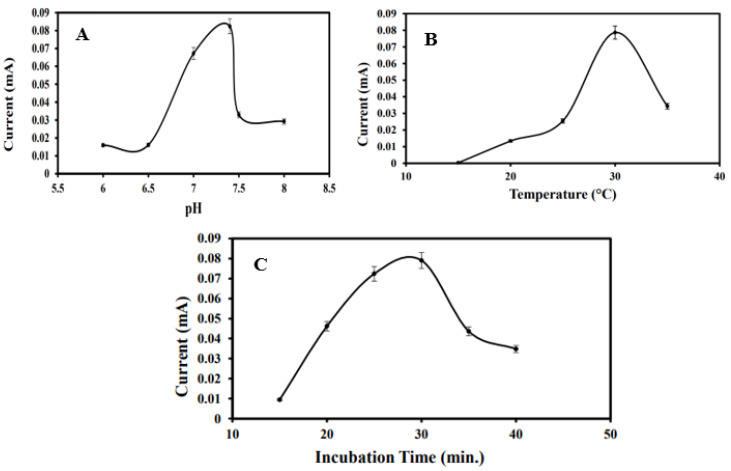
(**A**) Optimization graph of the optimization study of the immunosensor in the range of pH 6, pH 6.5, pH 7, pH 7.4, pH 7.5, and pH 8. (**B**) Temperature in the range of 15, 20, 25, 30, and 35 °C. (**C**) incubation time at 15, 20, 25, 30, 35, and 40 min.

The effect of pH on the electrochemical performance of the immunosensor was systematically investigated across a range of buffer pH values: 6.0, 6.5, 7.0, 7.4, 7.5, and 8.0. Electrodes modified with MoS_2_/CS/AuNPs/Anti-BRCA-1 were incubated in BRCA-1-containing PBS solutions at the specified pH levels, and the resulting current responses were recorded and plotted (Figure 4A). The signal intensity increased progressively up to pH 7.4, beyond which a decline was observed. Considering that the physiological pH is approximately 7.2, the optimal sensor response at pH 7.4 was deemed appropriate for subsequent analytical applications.

Temperature-dependent immunoreactivity between anti-BRCA-1 antibodies and BRCA-1 antigens was also evaluated within the range of 15 °C to 35 °C (Figure 4B). The most pronounced current response was achieved at 30 °C, which was selected as the optimal operating temperature. A reduction in signal at elevated temperatures was likely due to partial denaturation or conformational changes in the antibody molecules. Additionally, the ideal antigen–antibody interaction time was determined to be 30 min; extending incubation beyond this point yielded negligible improvements in the current response.

Furthermore, antibody immobilization time was optimized by exposing MoS_2_/CS/AuNP-modified PGEs to a 10 ng·mL^−1^ anti-BRCA-1 solution for durations ranging from 15 to 40 min. As depicted in Figure 4C, the current response increased up to 30 min and subsequently plateaued, suggesting saturation of the electrode surface. Therefore, an antibody immobilization time of 30 min was selected for further studies.

### 3.4. Analytical Performance of the Immunosensor

**Figure 5:**
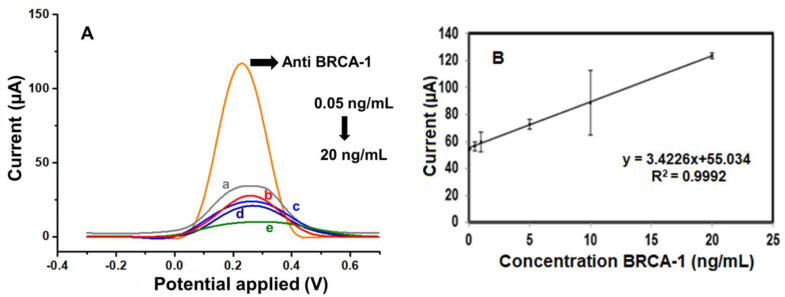
(**A**) DPV curves of the **a**. 0.05, **b**. 0.5, **c**. 1, **d**. 10, and **e**. 20 ng/mL BRCA-1 by using a redox probe solution. (**B**) Calibration graph of the BRCA-1 immunosensor toward different concentrations (from 0.05 to 20 ng/mL).

Following the determination of optimal experimental conditions, the analytical capabilities of the fabricated immunosensor were assessed by monitoring its differential pulse voltammetry (DPV) response across a range of BRCA-1 concentrations. As illustrated in Figure 5A, the sensor exhibited a consistent and concentration-dependent decrease in peak current over the 0.05 to 20 ng·mL^−1^ interval. A calibration graph (Figure 5B) was constructed by plotting the current responses against the logarithm of BRCA-1 concentration, yielding a linear trend described by the regression equation, I (μA) = 34.226 × log[BRCA-1 (ng·mL^−1^)] + 55.034, with a correlation coefficient (R^2^) of 0.9992, indicating excellent linearity.

The limit of detection (LOD) was estimated to be 0.04 ng·mL^−1^, based on the 3σ/slope criterion, while the limit of quantification (LOQ) was determined as 0.13 ng·mL^−1^ (10σ/slope). To evaluate the reproducibility of the sensor, three immunosensors were independently fabricated and tested under identical conditions. The calculated relative standard deviation (RSD) was 3.59%, confirming good fabrication reproducibility.

A comparative analysis with previously reported electrochemical platforms for BRCA-1 detection is presented in Table 1. The proposed sensor demonstrated favorable analytical performance in terms of detection limit, dynamic range, and operational simplicity, supporting its potential utility in clinical diagnostics for early cancer biomarker screening.

The observed characteristics underscore the heightened sensitivity and strong reproducibility of the immunosensor, as outlined in Table 1.

### 3.5. Selectivity, Stability, and Real Sample Analysis

The selectivity and clinical relevance of the developed electrochemical immunosensor were evaluated by analyzing its response to BRCA-1 in both phosphate-buffered saline (PBS) and human serum matrices. Given the distinct physicochemical properties and ionic content of these environments, measurements were carried out under identical optimized conditions using 10 ng·mL^−1^ BRCA-1 to assess matrix-dependent performance.

**Figure 6:**
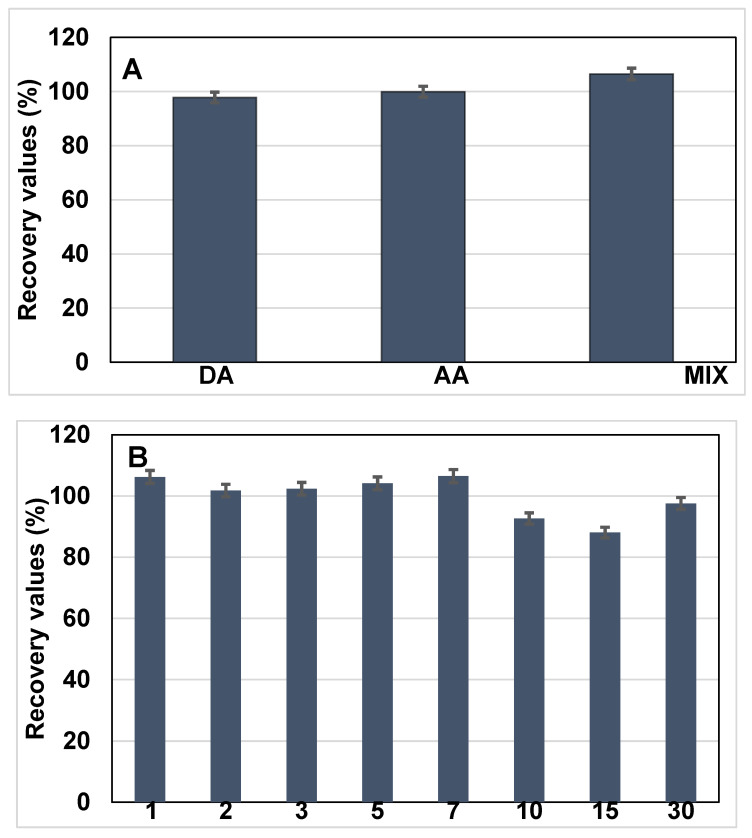
(**A**) Selectivity performances of the developed immunosensor in the detection of BRCA-1 in the presence of dopamine (DA), ascorbic acid (AA), and mix inhibitors. (**B**) Long-term stability of the electrode (*n* = 3).

To investigate potential cross-reactivity and sensor specificity, the sensor was further tested in the presence of electroactive interferents, namely dopamine (DA) and ascorbic acid (AA), each introduced at a concentration of 10 ng·mL^−1^ along with BRCA-1. The resulting current responses were monitored and compared with those obtained from BRCA-1 alone, as shown in Figure 6A.

Initially, BRCA-1 was introduced into serum, and the corresponding change in the current signal was recorded following incubation. In the subsequent experiment, a mixed solution containing BRCA-1, DA, and AA (each at 10 ng·mL^−1^) was applied to the immunosensor under identical conditions. The difference in current response between the two cases served as an indicator of the biosensor’s selectivity toward BRCA-1 in complex biological media.

The long-term operational stability of the fabricated immunosensor was assessed by conducting differential pulse voltammetry (DPV) analyses over a 30-day period. Measurements were systematically recorded on days 1, 2, 3, 5, 7, 10, 15, and 30 to monitor any signal variations during storage (Figure 6B). Throughout the study period, the electrodes were stored at 4 °C under dry conditions. The sensor maintained consistent electrochemical responses, indicating minimal degradation and favorable storage stability. On day 15, the relative standard deviation (RSD) for the current response was calculated as 6.48% based on triplicate measurements (*n* = 3), demonstrating acceptable reproducibility and reliable performance over time.

## 4. Conclusions

In conclusion, a novel electrochemical immunosensor was successfully fabricated utilizing a nanocomposite composed of molybdenum disulfide (MoS_2_), chitosan (CS), and gold nanoparticles (AuNPs) for the ultrasensitive detection of the BRCA-1 cancer biomarker. The combined physicochemical advantages of these materials provided a conducive microenvironment for effective antibody immobilization and facilitated enhanced electron transfer at the electrode interface. The resulting immunosensor demonstrated impressive analytical performance, featuring a broad linear detection range (0.05–20 ng·mL^−1^), a low detection limit of 0.04 ng·mL^−1^, and high reproducibility, with a relative standard deviation (RSD) of 3.59%. The system also exhibited excellent selectivity and operational stability across all tested conditions. For practical assessment, the device was evaluated in both human serum and phosphate-buffered saline (PBS), with comparative measurements indicating a strong correlation of 97 ± 3.5% between matrices. Furthermore, the immunosensor maintained specificity in the presence of potentially interfering analytes, such as dopamine and ascorbic acid, with negligible deviation from BRCA-1-only responses (98 ± 2.82%). Overall, the developed sensor showed consistent repeatability, stability, and selectivity, confirming its potential for real-world applications. Beyond cancer diagnostics, this electrochemical platform may serve as a versatile tool in areas such as biomarker research, enzyme activity monitoring, biosensor design, and electrochemical energy systems. The incorporation of emerging nanomaterials in future iterations could further enhance its performance and expand its application spectrum.

Moreover, the developed immunosensor exhibited reliable and consistent performance when applied to human serum samples, underscoring its feasibility for use in clinical diagnostics of cancer-related biomarkers. Beyond its immediate utility in BRCA-1 detection, this study offers a valuable framework for the design and optimization of highly sensitive electrochemical immunosensing platforms applicable to a broad spectrum of oncological markers.

## Figures and Tables

**Table 1:** LOD values and calibration constants.

Sensor Matrix	LOD	RSD	Slope	Intercept	R^2^	Concentration Range
PGE/MoS_2_/Cs/AuNp/Anti-BRCA-1/BSA/BRCA-1	0.04 ng/mL^−1^	3.59%	34.226	55.034	0.9992	0.05–20 ng/mL

[LOD = 3 × (std deviation of blank)/(slope of calibration)]; std deviation of blank was calculated for three measurements [32].

## Data Availability

The original contributions presented in the study are included in the article; further inquiries can be directed to the corresponding author.
